# EQ-5D-5L-based quality of life normative data for patients with self-reported diabetes in Poland

**DOI:** 10.1371/journal.pone.0257998

**Published:** 2021-09-29

**Authors:** Agnieszka Jankowska, Dominik Golicki

**Affiliations:** 1 National Institute of Cardiology, Warsaw, Poland; 2 Department of Experimental and Clinical Pharmacology, Medical University of Warsaw, Warsaw, Poland; Chiang Mai University Faculty of Medicine, THAILAND

## Abstract

**Introduction:**

The new, five-level EQ-5D generic questionnaire (EQ-5D-5L) has never been used among diabetes patients in Poland.

**Objectives:**

To develop health-related quality of life (HRQoL) norms for patients with self-reported diabetes, based on a large representative sample of the general Polish population, using the EQ-5D-5L.

**Materials and methods:**

Members of the general public, selected via multistage stratified sampling, filled in the EQ-5D-5L questionnaire and answered a question about the presence of diabetes. We estimated three types of EQ-5D-5L outcomes: limitations within domains, EQ VAS and EQ-5D-5L index. Multiple linear regression was used to examine the relationship between sociodemographic characteristics and HRQoL, both in patients with diabetes and the general population sample.

**Results:**

Among 2,973 respondents having complete EQ-5D-5L data, 255 subjects (8.6%) self-reported diabetes. Treatment with insulin, other drugs, combination therapy or lack of drug treatment was declared by 22.0%, 48.6%, 5.1% and 24.3% of patients, respectively. Respondents with diabetes had a lower EQ VAS score (18.5 points difference on a 100-points scale) and a lower EQ-5D-5L index score (0.135 difference; scale range: 1.59). The multivariate analysis showed that the factors independently improving the HRQoL in the general population were secondary or higher education, and factors reducing HRQoL were female sex, belonging to an older age group, being treated because of diabetes with insulin, other drugs or combination treatment. Respondents diagnosed with diabetes but not treated with drugs showed a decrease in EQ VAS scores, but not in the EQ-5D-5L index.

**Conclusions:**

Diabetes leads to HRQoL deterioration in all age groups when compared to matched general population respondents without diabetes. The most significant HRQoL reduction experience older patients with a basic level of education. Obtained EQ-5D-5L normative data may be used in the clinical care of patients with diabetes and health technology assessment of new anti-diabetic drugs.

## Introduction

Diabetes mellitus (DM) is a public health problem, particularly in highly developed countries. The International Diabetes Federation estimates the global number of patients with diabetes will exceed 700 million by the year 2030 [1]. A study on disease burdens showed that in Poland, the direct costs of diabetes treatment doubled in the 2005–2009 period [2]. It is estimated that during the years 2012–2014 in Poland, diabetes and its complications were responsible for over 82,000 lost working years, which resulted in over USD 1.9 billion of total indirect costs [3].

DM type 2 is a civilization disease. If the condition is not well controlled with the available treatments, DM leads to severe macro- and microvascular complications and results in increased mortality and reduction of quality of life. Health-related quality of life (HRQoL) in diabetes patients can be measured with numerous disease-specific questionnaires [4–13]. Researchers interested in selected areas may use even more specific instruments—on adherence to treatment [14], emotional stress [15, 16], knowledge about diabetes [17, 18], self-efficacy [19–21], evaluation of hypoglycaemia [22, 23] or focused on specific subgroups of patients [24].

An alternative approach to measuring HRQoL in patients with diabetes is based on the use of generic instruments, which, by definition, apply to the general population, as well as to a variety of health states, conditions and diseases. Whereas the most popular health profile seems to be the Medical Outcomes Study Short Form-36 (SF-36) [25], the most commonly used preference-based measure is undoubtedly the instrument developed by the EuroQol Group–the EQ-5D [26]. The three-level version of the latter (EQ-5D-3L) has been extensively used in patients with diabetes [27]. Recent years have brought the development of a five-level version of the EQ-5D (EQ-5D-5L), which is characterized by improved psychometric properties [28]. Use of generic questionnaires, such as EQ-5D, enables comparison of patient groups with the general population of the country, and objective assessment of the burden of disease.

Our study aimed to develop quality of life normative data for patients with self-reported diabetes based on a large, representative sample of the general Polish population, with the use of the EQ-5D-5L questionnaire.

## Materials and methods

### Study design and sample

The study was a cross-sectional survey, performed with the use of multistage random sampling. Sample recruitment was carried out by a market research company—Public Opinion Research Center (CBOS). To obtain a representative study group, taking into account the country’s administrative division (16 ‘voivodeships’ or provinces) and the type and size of localities in each province, the Polish adult population was divided into 65 strata. The predetermined study sample was proportionally allocated into layers, so as to reflect the general population structure. Multistage random sampling was carried out at three successive levels of granularity: (1) towns/cities and villages; (2) small areas (one or several adjacent streets) within the previously drawn localities; (3) according to the Polish Resident Identification Number (PESEL)—a sample of eight people living in separate dwelling/household from each of the selected areas.

The need for ethics approval for this study was waived by the Bioethical Commission of the Medical University of Warsaw (AKBE/95/2019). Written informed consent was not required for participation in the study. Oral consent was obtained. The data were analyzed anonymously.

### Survey

The survey consisted of three sections in the following order: (I) sociodemographic questions, (II) self-reported presence of diabetes and (III) quality of life section (EQ-5D-5L, SF-12 and EQ-5D-3L questionnaires). In the current paper we are focusing on EQ-5D-5L results. The SF-12 and EQ-5D-3L outcomes were described elsewhere [29, 30]. The current study was run as a part of a larger survey (an Omnibus study).

Sociodemographic questions covered the following: type of locality, voivodeship (province), level of education, occupational status, household income, religiosity and smoking habits.

We classified respondents as having self-reported diabetes if, in response to the following question: “Have you ever been diagnosed with diabetes?”, they chose one of the following answers: (1) “Yes, but I don’t take any medication”, (2) “Yes, I take anti-diabetic medication (other than insulin)” or (3) “Yes, I take insulin”. Respondents were allowed to choose both answers (2) and (3) when they were on combined treatment.

The study used the EQ-5D-5L questionnaire, which consists of two parts: a descriptive system and a visual analog scale (EQ VAS) [31]. The descriptive part comprises five dimensions: mobility (MO), self-care (SC), usual activities (UA), pain/discomfort (PD) and anxiety/depression (AD). Each of the EQ-5D-5L items has five possible levels, of which four are common to all dimensions: (1) no problems, (2) slight problems, (3) moderate problems and (4) serious problems. The fifth answer for the dimensions MO, SC and UA was formulated as incapacity, and for PD and AD as an extreme feeling. Five scales with five possible answers result in a total of 3,125 possible health states.

Additionally, based on the respondent’s answers, a weighted measure of health may be calculated—EQ-5D index. It is used in pharmacoeconomics and health technology assessment to calculate quality-adjusted life years (QALY) [32]. The EQ-5D-5L Index value scale extends from ‘1’, for perfect health, through to ‘0’, which corresponds to the death state, and on to negative values, which indicate states even worse than death, according to the perceptions of a given society. For the assessment of EQ-5D-5L index, Polish directly measured, time trade-off (TTO) and discrete choice experiment (DCE)-based, EQ-5D-5L value set was used [33].

EQ VAS is a visual analog scale, where values from 0 to 100 appear on a 20 cm vertical axis, where 0 means ‘the worst imaginable health state’ and 100 means ‘the best imaginable health state’. It constitutes a subjective measure of health.

### Data collection

The data were collected by professional CBOS interviewers during face-to-face interviews (April to June 2014). The EQ-5D-5L questionnaire was distributed as a paper-and-pencil version. This distribution method has predominantly been used in HRQoL studies in Poland until the present time. All other data were collected using the computer-assisted personal interviewing (CAPI) system.

### Data analysis

Results were presented for the whole sample, as well as for the predefined comparisons: (1) respondents with diabetes versus respondents without diabetes; (2) treated for diabetes versus untreated; (3) treated with insulin versus treated with other drugs versus treated with combined treatment. The mean values with standard deviation, median, interquartile range and range were estimated for the continuous variables, such as EQ VAS and EQ-5D-5L index. The distribution of answers to the questions in the descriptive part of the EQ-5D-5L was computed.

### Statistical analysis

Confidence intervals for proportions were calculated using the Clopper-Pearson method. The parametricity of the distribution was explored with the Shapiro-Wilk test. The statistical significance of differences in dichotomous variables was examined using Fisher’s exact test and in nominal variables by using a chi-square test. The Mann-Whitney test and ANOVA were used to assess differences between two and several demographic groups, respectively, in interval data, such as the EQ-5D index or EQ VAS. We used multiple linear regression to examine the associations of sociodemographic characteristics with the EQ-5D-5L index and EQ VAS scores, both in the population of diabetic patients and the whole population or respondents. All variables, including age, were entered into the models as categorical variables. We presented the regression coefficients, together with information about the level of statistical significance. The analysis was conducted using StatsDirect 3.1.22 statistical software (StatsDirect Ltd, Altrincham, England).

## Results

### Studied population

The current analysis is based on data from 2,973 (99.6%) respondents (age range 18–87 years, 46.8% men, 36.7% inhabitants of rural areas), out of 2,986, for which complete answers to the EQ-5D-5L questionnaire were available (Table 1).

**Table 1:** Characteristics of respondents according to diabetes and treatment status.

	All	Diabetes			Drug treatment			Type of treatment		
		No	Yes	p	No	Yes	p	Drugs other than insulin	Insulin	Combination treatment
**N**	2973	2718	255		62	193		124	56	13
**Gender, n (%)**										
Female	1583 (53.2)	1444 (53.1)	139 (54.5)	NS	32 (51.6)	86 (44.6)	NS	68 (54.8)	30 (53.6)	9 (69.2)
Male	1390 (46.8)	1274 (46.9)	116 (45.5)		30 (48.4)	107 (55.4)		56 (45.2)	26 (46.4)	4 (30.8)
**Age, mean (SD)**	48.6 (17.9)	47.1 (17.7)	64.6 (12.1)	0.0001	57.0 (16.6)	67.1 (9.1)	0.0001	67.0 (8.9)	67.6 (9.3)	65.2 (9.9)
**Place of residence, n (%)**										
Village	1090 (36.7)	1009 (37.1)	81 (31.8)	NS	18 (29.0)	63 (32.6)	NS	38 (30.6)	18 (32.1)	7 (53.8)
Town up to 20,000	505 (17.0)	456 (16.8)	49 (19.2)		13 (21.0)	36 (18.7)		29 (23.4)	7 (12.5)	0 (0)
Town 20,000–49,999	335 (11.27)	301 (11.1)	34 (13.3)		12 (19.4)	22 (11.4)		15 (12.1)	5 (8.9)	2 (15.4)
Town 50,000–99,999	236 (7.9)	218 (8.0)	18 (7.1)		1 (1.6)	17 (8.8)		10 (8.1)	5 (8.9)	2 (15.4)
Town 100,000–499,999	507 (17.1)	459 (16.9)	48 (18.8)		12 (19.4)	36 (18.7)		24 (19.4)	11 (19.6)	1 (7.7)
Town ≥500,000	300 (10.1)	275 (10.1)	25 (9.8)		6 (9.7)	19 (9.8)		8 (6.5)	10 (17.9)	1 (7.7)
**Region, n (%)**										
Lower Silesia Province	262 (8.8)	240 (8.8)	22 (8.6)	NS	6 (9.7)	16 (8.3)	NS	10 (8.1)	6 (10.7)	0 (0)
Kujawy-Pomerania Province	149 (5.0)	137 (5.0)	12 (4.7)		2 (3.2)	10 (5.2)		9 (7.3)	1 (1.8)	0 (0)
Lublin Province	153 (5.1)	141 (5.2)	12 (4.7)		5 (8.1)	7 (3.6)		6 (4.8)	1 (1.8)	0 (0)
Lubuskie Province	76 (2.6)	64 (2.4)	12 (4.7)		1 (1.6)	11 (5.7)		7 (5.6)	4 (7.1)	0 (0)
Łódź Province	209 (7.0)	198 (7.3)	11 (4.3)		2 (3.2)	9 (4.7)		6 (4.8)	3 (5.4)	0 (0)
Małopolska Province	277 (9.3)	252 (9.3)	25 (9.8)		5 (8.1)	20 (10.4)		11 (8.9)	9 (16.1)	0 (0)
Mazovia Province	377 (12.7)	347 (12.8)	30 (11.8)		9 (14.5)	21 (10.9)		13 (10.5)	7 (12.5)	1 (7.7)
Opole Province	77 (2.6)	69 (2.5)	8 (3.1)		4 (6.5)	4 (2.1)		3 (2.4)	1 (1.8)	0 (0)
Podkarpackie Province	173 (5.8)	154 (5.7)	19 (7.5)		4 (6.5)	15 (7.8)		12 (9.7)	3 (5.4)	0 (0)
Podlasie Province	103 (3.5)	94 (3.5)	9 (3.5)		2 (3.2)	7 (3.6)		4 (3.2)	2 (3.6)	1 (7.7)
Pomerania Province	151 (5.1)	135 (5.0)	16 (6.3)		1 (1.6)	15 (7.8)		9 (7.3)	5 (8.9)	1 (7.7)
Silesia Province	395 (13.3)	364 (13.4)	31 (12.2)		8 (12.9)	23 (11.9)		13 10.5)	7 (12.5)	1 (7.7)
Świętokrzyskie Province	93 (3.1)	89 (3.3)	4 (1.6)		1 (1.6)	3 (1.6)		1 (0.8)	2 (3.6)	0 (0)
Warmia-Masuria Province	101 (3.4)	92 (3.4)	9 (3.5)		3 (4.8)	6 (3.1)		5 (4.0)	1 (1.8)	0 (0)
Wielkopolska Province	247 (8.3)	225 (8.3)	22 (8.6)		5 (8.1)	17 (8.8)		9 (7.3)	4 (7.1)	4 (30.8)
West-Pomerania Province	130 (4.4)	117 (4.3)	13 (5.1)		4 (6.5)	9 (4.7)		6 (4.8)	0 (0)	3 (23.1)
**Education level, n (%)**										
Low	1268 (42.7)	1134 (41.7)	134 (52.5)	0.001	33 (53.2)	101 (52.3)	NS	59 (47.6)	36 (64.3)	6 (46.2)
Medium	1089 (36.6)	1001 (36.8)	88 (34.5)		18 (29.0)	70 (36.3)		50 (40.3)	16 (28.6)	4 (30.8)
High	616 (20.7)	583 (21.4)	33 (12.9)		11 (17.7)	22 (11.4)		15 (12.1)	4 (7.1)	3 (23.1)
**Occupational status, n (%)**										
Employed	1451 (48.8)	1399 (51.5)	52 (20.4)	0.0001	25 (40.3)	27 (14.0)	0.0001	18 (14.5)	8 (14.3)	1 (7.7)
Unemployed	203 (6.8)	196 (7.2)	7 (2.7)		3 (4.8)	4 (2.1)		2 (1.6)	1 (1.8)	1 (7.7)
Retired	948 (31.9)	763 (28.1)	185 (72.5)		30 (48.4)	155 (80.3)		98 (79.0)	46 (82.1)	11 (84.6)
Student	208 (7.0)	207 (7.6)	1 (0.4)		1 (1.6)	0 (0)		0 (0)	0 (0)	0 (0)
Domestic	106 (3.6)	100 (3.7)	6 (2.4)		2 (3.2)	4 (2.1)		4 (3.2)	0 (0)	0 (0)
Other	57 (1.9)	53 (1.9)	4 (1.6)		1 (1.6)	3 (1.6)		2 (1.6)	1 (1.8)	0 (0)
**Smoking status, n (%)**										
Yes	777 (26.1)	721 (26.5)	56 (22.0)	0.0001	13 (21.0)	43 (22.3)	NS	22 (17.7)	17 (30.4)	4 (30.8)
No	1689 (56.8)	1564 (57.5)	125 (49.0)		33 (53.2)	92 (47.7)		64 (51.6)	24 (42.9)	4 (30.8)
In the past	492 (16.5)	418 (15.4)	74 (29.0)		16 (25.8)	58 (30.1)		38 (30.6)	15 (26.8)	5 (38.5)
**Net monthly income of household (PLN), mean (SD)**	1396 (1048)	1396 (1078)	1407 (755)	0.06	1409 (802)	1406 (744)	NS	1402 (713)	1353 (824)	1820 (706)

NS, non-significant.

In the analysed population, the diagnosis of diabetes was declared by 255 subjects, which corresponds to a prevalence of diabetes at a level of 8.6% (95% CI 7.6–9.6). A lack of drug treatment was present for a significant percentage of respondents—24.3% (95% CI 19.2–30.1). Patients treated with drugs other than insulin, insulin itself or a combination therapy constituted 48.6% (95% CI 42.3–54.9), 22.0% (95% CI 17.9–27.5) and 5.1% (95% CI 2.7–8.6) respectively of respondents with self-reported diabetes, and 64.3% (95% CI 57.0–71.0), 29.0% (95% CI 22.7–36.0) and 6.7% (95% CI 3.6–11.2) of respondents declaring diabetes drug treatment.

Respondents with diabetes, compared to respondents without diabetes, were older (average age difference—17.5 years) and were characterized by a lower level of education, lower employment rates (20.4% vs 51.5%), a higher percentage of pensioners (72.5% vs 28.1%), former smokers (29.0% vs 15.4%) and people reporting health limitations based on the EQ-5D-5L questionnaire (90.6% vs 58.0%).

Patients with treated diabetes, compared to untreated, were also older (mean age of 67.1 vs 57.0), more often retired, less likely to be working and with more frequently reported health problems according to the EQ-5D questionnaire (93.8% vs 80.6%).

### EQ-5D-5L dimensions

Table 2 presents the level of problems in diabetes patients according to the EQ-5D-5L dimensions.

**Table 2:** Problems in EQ-5D-5L dimensions according to diabetes and treatment status, n (%).

	All	Diabetes			Drug treatment			Type of treatment		
		No	Yes	p	No	Yes	p	Drugs other than insulin	Insulin	Combination treatment
**N**	2973	2718	255		62	193		124	56	13
**Health state according to EQ-5D-5L, n (%)**										
No problems (11111)	1165 (39.2)	1141 (42.0)	24 (9.4)	0.0001	12 (19.4)	12 (6.2)	0.01	10 (8.1)	2 (3.6)	0 (0)
Any problems	1808 (60.8)	1577 (58.0)	231 (90.6)		50 (80.6)	181 (93.8)		114 (91.9)	54 (96.4)	13 (100)
**Mobility**										
No problems	2190 (73.7)	2093 (77.0)	97 (38.0)	0.001	34 (54.8)	63 (32.6)	0.01	43 (34.7)	17 (30.4)	3 (23.1)
Slight problems	340 (11.4)	286 (10.5)	54 (21.2)		11 (17.7)	43 (22.3)		29 (23.4)	9 (16.1)	5 (38.5)
Moderate problems	223 (7.5)	179 (6.6)	44 (17.3)		11 (17.7)	33 (17.1)		23 (18.5)	9 (16.1)	1 (7.7)
Severe problems	201 (6.8)	150 (5.5)	51 (20.0)		5 (8.1)	46 (23.8)		24 (19.4)	18 (32.1)	4 (30.8)
Incapacity	19 (0.6)	10 (0.4)	9 (3.5)		1 (1.6)	8 (4.1)		5 (4.0)	3 (5.4)	0 (0)
**Self-care**										
No problems	2701 (90.6)	2512 (92.4)	189 (74.1)	0.001	49 (79.0)	140 (72.5)	NS	93 (75.0)	37 (66.1)	10 (76.9)
Slight problems	125 (4.2)	99 (3.6)	26 (10.2)		6 (9.7)	20 (10.4)		11 (8.9)	7 (12.5)	2 (15.4)
Moderate problems	95 (3.2)	70 (2.6)	25 (9.8)		6 (9.7)	19 (9.8)		12 (9.7)	6 (10.7)	1 (7.7)
Severe problems	40 (1.3)	30 (1.1)	10 (3.9)		0 (0)	10 (5.2)		5 (4.0)	5 (8.9)	0 (0)
Incapacity	12 (0.4)	7 (0.3)	5 (2.0)		1 (1.6)	4 (2.1)		3 (2.4)	1 (1.8)	0 (0)
**Usual activities**										
No problems	2456 (82.6)	2305 (84.8)	151 (59.2)	0.001	44 (71.0)	107 (55.4)	0.04	76 (61.3)	24 (42.9)	7 (53.8)
Slight problems	275 (9.2)	232 (8.5)	43 (16.9)		10 (16.1)	33 (17.1)		18 (14.5)	13 (23.2)	2 (15.4)
Moderate problems	150 (5.0)	114 (4.2)	36 (14.1)		5 (8.1)	31 (16.1)		21 (16.9)	7 (12.5)	3 (23.1)
Severe problems	76 (2.6)	59 (2.2)	17 (6.7)		2 (3.2)	15 (7.8)		6 (4.8)	9 (16.1)	0 (0)
Incapacity	16 (0.5)	8 (0.3)	8 (3.1)		1 (1.6)	7 (3.6)		3 (2.4)	3 (5.4)	1 (7.7)
**Pain/discomfort**										
No	1438 (48.4)	1391 (51.2)	47 (18.4)	0.001	21 (33.9)	26 (13.5)	0.001	18 (14.5)	7 (12.5)	1 (7.7)
Slight	795 (26.7)	726 (26.7)	69 (27.1)		19 (30.6)	50 (25.9)		32 (25.8)	15 (26.8)	3 (23.1)
Moderate	513 (17.3)	430 (15.8)	83 (32.5)		15 (24.2)	68 (35.2)		48 (38.7)	15 (26.8)	5 (38.5)
Severe	211 (7.1)	162 (6.0)	49 (19.2)		6 (9.7)	43 (22.3)		23 (18.5)	16 (28.6)	4 (30.8)
Extreme	16 (0.5)	9 (0.3)	7 (2.7)		1 (1.6)	6 (3.1)		3 (2.4)	3 (5.4)	0 (0)
**Anxiety/depression**										
No	1748 (58.8)	1666 (61.3)	82 (32.2)	0.001	23 (37.1)	59 (30.6)	NS	37 (29.8)	19 (33.9)	3 (23.1)
Slight	806 (27.1)	713 (26.2)	93 (36.5)		28 (45.2)	65 (33.7)		43 (34.7)	18 (32.1)	4 (30.8)
Moderate	310 (10.4)	258 (9.5)	52 (20.4)		8 (12.9)	44 (22.8)		28 (22.6)	11 (19.6)	5 (38.5)
Severe	96 (3.2)	71 (2.6)	25 (9.8)		2 (3.2)	23 (11.9)		14 (11.3)	8 (14.3)	1 (7.7)
Extreme	13 (0.4)	10 (0.4)	3 (1.2)		1 (1.6)	2 (1.0)		2 (1.6)	0 (0)	0 (0)

NS, non-significant.

In general, patients with diabetes were characterized by a similar picture of the affected domains to that of the general population or to respondents without diabetes (dimensions in order from most to least affected being: PD, AD, MO, UA, SC). The identical pattern was typical for both untreated and treated diabetes, and it only changed in the subpopulation having insulin treatment, where the number of MO health limitations exceeded that in the AD dimension.

In terms of all the domains in the EQ-5D-5L questionnaire, diabetes respondents had a higher frequency of restrictions compared to both the general and non-diabetic populations. The most significant differences in the prevalence of any problems concerned MO, PD and AD—with 35.7%, 30.0% and 26.5% more restrictions, respectively, compared to the entire study population, and 39.0%, 32.8% and 29.0% more than the non-diabetic population.

Treated diabetes patients, as compared to non-treated, had a statistically significant higher incidence of restrictions within MO, PD and UA. At the type of therapy level, in terms of SC and UA, insulin-treated patients had the most problems, whereas in terms of MO, PD and AD it was those treated with a combination therapy.

### EQ-5D-5L health states in patients with diabetes

In the 255 respondents with diabetes, 121 different EQ-5D-5L health states were identified, including 39 that occurred in at least two respondents and 8 that occurred in at least five (Table 3). The most common health condition declared was 11111 - ‘without any limitations’ (n = 24; 9.4%), followed by 11122 (n = 17; 6.7%).

**Table 3:** Diabetes patients’ health status according to EQ-5D-5L (N = 255).

EQ-5D-5L health state	Frequency, n	Relative frequency (%)	EQ-5D-5L health state	Frequency, n	Relative frequency (%)
11111	24	9.4	21133	2	0.8
11122	17	6.7	21141	2	0.8
11121	13	5.1	21142	2	0.8
11112	12	4.7	21221	2	0.8
21122	11	4.3	21222	2	0.8
11131	8	3.1	21232	2	0.8
21132	7	2.7	22221	2	0.8
21121	6	2.4	22222	2	0.8
11133	4	1.6	31121	2	0.8
31132	4	1.6	31131	2	0.8
31133	4	1.6	32232	2	0.8
33333	4	1.6	33443	2	0.8
41232	4	1.6	41131	2	0.8
11123	3	1.2	41143	2	0.8
21112	3	1.2	41243	2	0.8
31233	3	1.2	42343	2	0.8
11114	2	0.8	43332	2	0.8
11132	2	0.8	43333	2	0.8
11221	2	0.8	44442	2	0.8
21131	2	0.8			

### EQ VAS

Subjective health assessment (EQ VAS) was significantly lower in respondents with diabetes compared to non-diabetic population—a difference of 18.5 points (scale range 100 points; p <0.0001; Table 4). In diabetes patients, the subjective assessment of health was lower in treated respondents than non-treated—a difference of 8.6 points (p <0.01). A lower EQ VAS value was also observed in patients on insulin therapy versus those treated with other drugs—a difference of 8.9 points (p <0.05). The highest EQ VAS values were recorded in patients with diabetes belonging to the youngest age group of 18–49 years (69.4). They were significantly lower in the age groups of 50–64 years and above 65 years, with values of 58.7 and 52.8 respectively (p <0.001; Table 5).

**Table 4:** EQ VAS and EQ-5D-5L index according to diabetes and treatment status.

EQ-5D-5L outcome	All	Diabetes			Drug treatment			Type of treatment			
		No	Yes	p	No	Yes	p	Drugs other than insulin	Insulin	Combination treatment	p
**N**	2973	2718	255		62	193		124	56	13	
**EQ VAS**											
mean (SD)	73.5 (20.1)	75.1 (19.2)	56.6 (21.5)	<0.0001	63.0 (20.7)	54.4 (21.4)	<0.01	57.4 (21.6)	48.5 (21.0)	51.9 (16.8)	<0.05 oral drugs vs insulin
median (Q1-Q3)	80 (90–60)	80 (90–60)	50 (70–50)		60 (80–50)	50 (70–45)		60 (70–50)	50 (60–40)	50 (55–50)	
min/max	0/100	0/100	0/100		10/100	0/100		0/100	0/90	20/90	
**EQ-5D-5L index**											
mean (SD)	0.920 (0.150)	0.932 (0.130)	0.797 (0.251)	<0.0001	0.874 (0.208)	0.772 (0.259)	<0.0001	0.795 (0.267)	0.719 (0.271)	0.781 (0.213)	<0.05 oral drugs vs insulin
median (Q1-Q3)	0.970 (1–0.922)	0.970 (1–0.929)	0.904 (0.952–0.714)		0.950 (0.982–0.864)	0.887 (0.945–0.685)		0.903 (0.950–0.720)	0.794 (0.941–0.573)	0.898 (0.972–0.685)	
min/max	-0.590/1	-0.590/1	-0.466/1		-0.387/1	-0.466/1		-0.466/1	-0.170/1	0.300/0.982	

NS, non-significant.

**Table 5:** Relation of EQ-5D-5L index and EQ VAS with demographic characteristics of diabetes patients (n = 255).

	N (%)	EQ-5D-5L index		EQ VAS	
		Mean (SD)	Multiple linear regression coefficients	Mean (SD)	Multiple linear regression coefficients
**Overall**	255	0.797 (0.251)		56.6 (21.5)	
**Intercept**			0.9117		74.64
**Gender**					
Male	116 (45.7)	0.829 (0.193)	-	59.4 (20.7)	-
Female	139 (54.5)	0.770 (0.289)	-0.0284	54.2 (22.0)	-3.86
**Age group**					
18–49 years	25 (9.8)	0.911 (0.124)	-	69.4 (24.3)	-
50–64 years	91 (35.7)	0.835 (0.219)	-0.0641	58.7 (21.4)	-11.3 *
65+ years	139 (54.5)	0.751 (0.277)	-0.1413 *	52.8 (20.1)	-17.2 *
**Place of residence**					
Country	81 (31.8)	0.784 (0.263)	-	57.3 (21.7)	-
City	174 (68.2)	0.802 (0.246)	-0.0212	56.2 (21.5)	-3.16
**Region**					
Lower Silesia Province	22 (8.6)	0.858 (0.198)	-	53.0 (19.8)	-
Kujawy-Pomerania Province	12 (4.7)	0.865 (0.125)	-0.0156	57.5 (13.1)	2.35
Lublin Province	12 (4.7)	0.746 (0.271)	-0.1337	60.3 (29.4)	4.73
Lubuskie Province	12 (4.7)	0.780 (0.182)	-0.0498	48.8 (15.4)	-1.92
Łódź Province	11 (4.3)	0.832 (0.154)	0.0025	60.5 (16.7)	9.20
Małopolska Province	25 (9.8)	0.837 (0.195)	-0.0168	60.2 (24.0)	6.68
Mazovia Province	7 (2.7)	0.793 (0.241)	-0.0704	58.3 (22.6)	3.59
Opole Province	8 (3.1)	0.928 (0.093)	0.0561	58.1 (12.5)	2.43
Podkarpackie Province	9 (3.5)	0.784 (0.171)	-0.0903	49.5 (17.6)	-5.51
Podlasie Province	9 (3.5)	0.570 (0.381)	-0.2710 *	48.9 (27.2)	-3.90
Pomerania Province	16 (6.3)	0.679 (0.446)	-0.1478 *	53.1 (29.4)	2.57
Silesia Province	31 (12.2)	0.790 (0.345)	-0.0686	61.9 (18.6)	8.43
Świętokrzyskie Province	13 (5.1)	0.871 (0.212)	-0.0400	61.3 (25.9)	3.05
Warmia-Masuria Province	14 (5.5)	0.800 (0.185)	-0.0450	55.0 (25.5)	1.88
Wielkopolska Province	22 (8.6)	0.783 (0.190)	-0.0711	52.1 (23.3)	-2.47
West-Pomerania Province	13 (5.1)	0.849 (0.197)	0.0035	64.5 (17.7)	11.70
**Education level**					
Low	64 (25.1)	0.696 (0.280)	-	49.1 (21.5)	-
Medium	148 (58.0)	0.819 (0.249)	0.1077 *	58.7 (21.4)	7.94 *
High	43 (16.9)	0.871 (0.154)	0.1182 *	60.3 (19.6)	8.39 *
**Smoking status**					
Yes	56 (22.0)	0.802 (0.276)	-	55.8 (22.8)	-
No	199 (78.0)	0.795 (0.245)	0.0347	56.8 (21.2)	4.12
**Religion**					
Believes	241 (94.5)	0.797 (0.253)	-	57.0 (21.3)	-
Don’t believe	14 (5.5)	0.800 (0.222)	-0.0192	48.9 (24.0)	-9.18

* Coefficients with the statistical significance (p <0.05)

### EQ-5D-5L index

**Fig 1:**
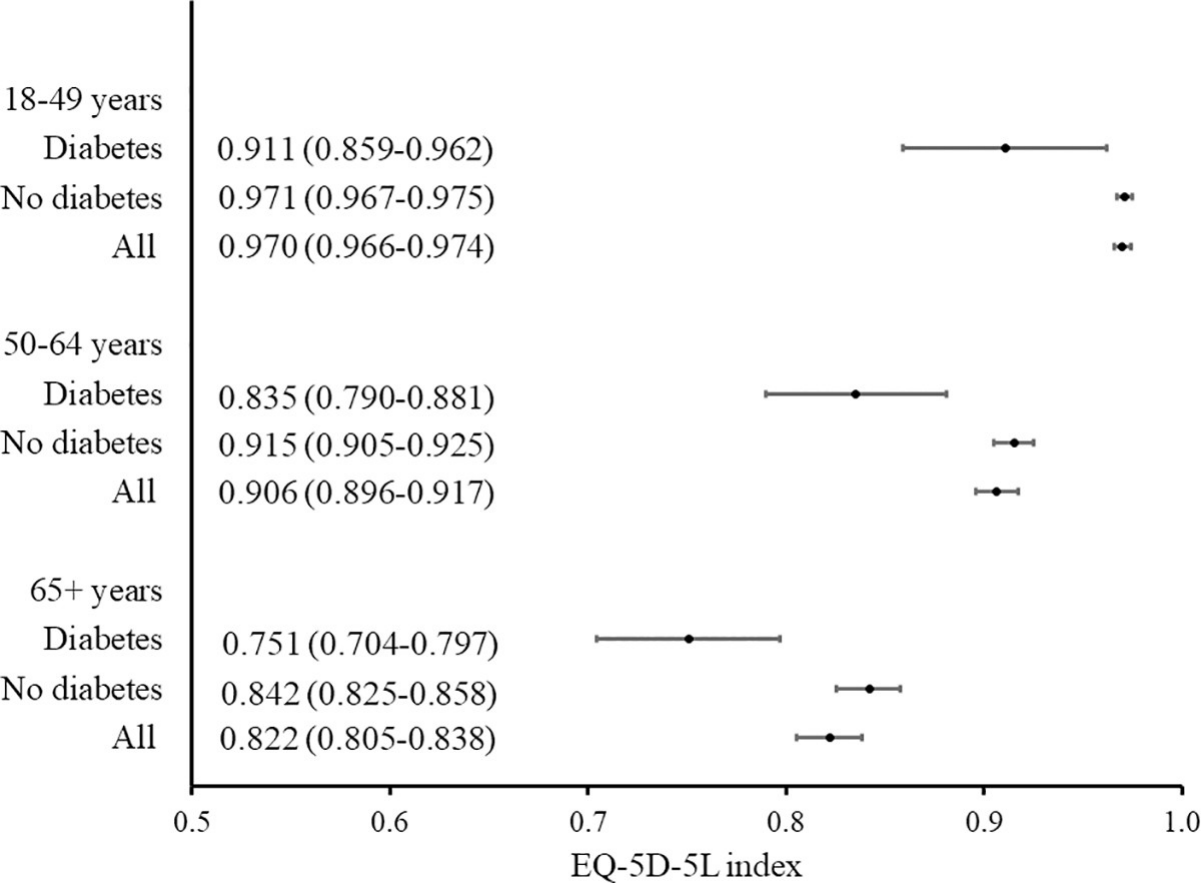
EQ-5D-5L index (mean, 95% confidence interval) according to age group: Comparison of respondents with diabetes versus no diabetes and the general population.

The results of the assessment of health, adjusted by the health preferences among Polish society (the Polish tariff-based EQ-5D-5L index) were consistent with the unweighted results and the subjective assessment. Respondents with diabetes, compared to non-diabetic ones, had a lower EQ-5D-5L value by an average of 0.135 (scale range: 1.59; p <0.0001). A similar result was observed for treated diabetic patients compared to untreated (difference of 0.102; p <0.0001) and patients treated with insulin compared to those taking other drugs (0.076 difference; p <0.05; Table 4). Higher EQ-5D-5L index values were characterized by patients with diabetes in younger age groups and with higher levels of education (Table 5, Fig 1).

### Sociodemographic characteristics and HRQoL in patients with diabetes

**Fig 2:**
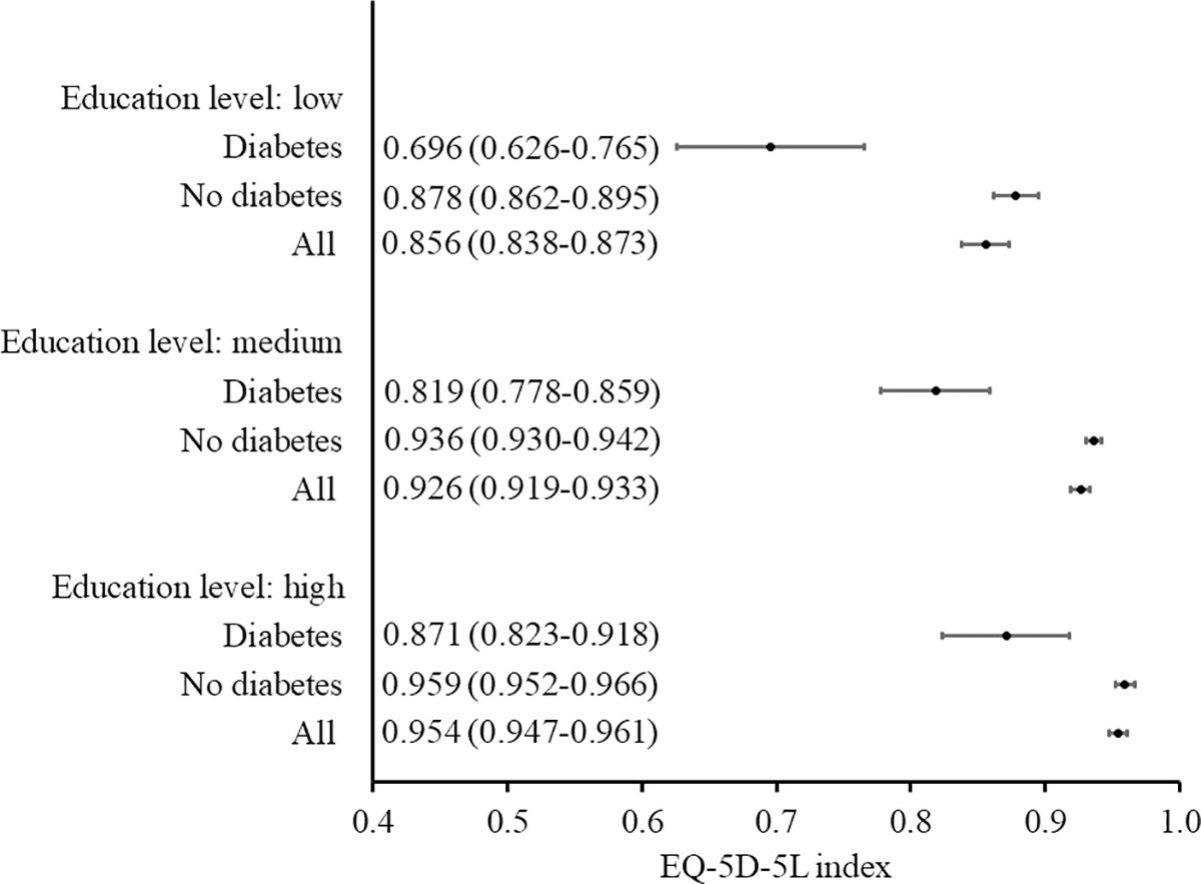
EQ-5D-5L index (mean, 95% confidence interval) according to education level: Comparison of respondents with diabetes versus no diabetes and the general population.

**Fig 3:**
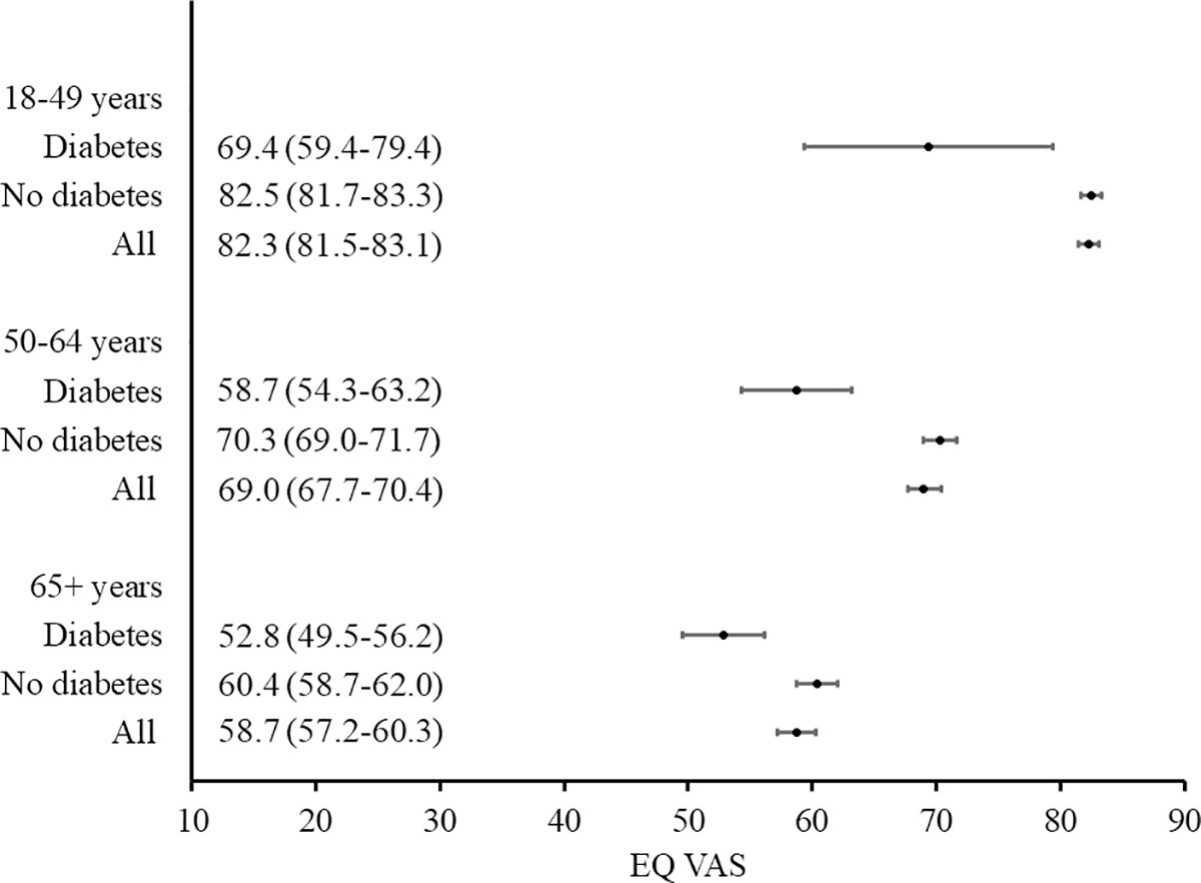
EQ VAS (mean, 95% confidence interval) according to age group: Comparison of respondents with diabetes versus no diabetes and the general population.

**Fig 4:**
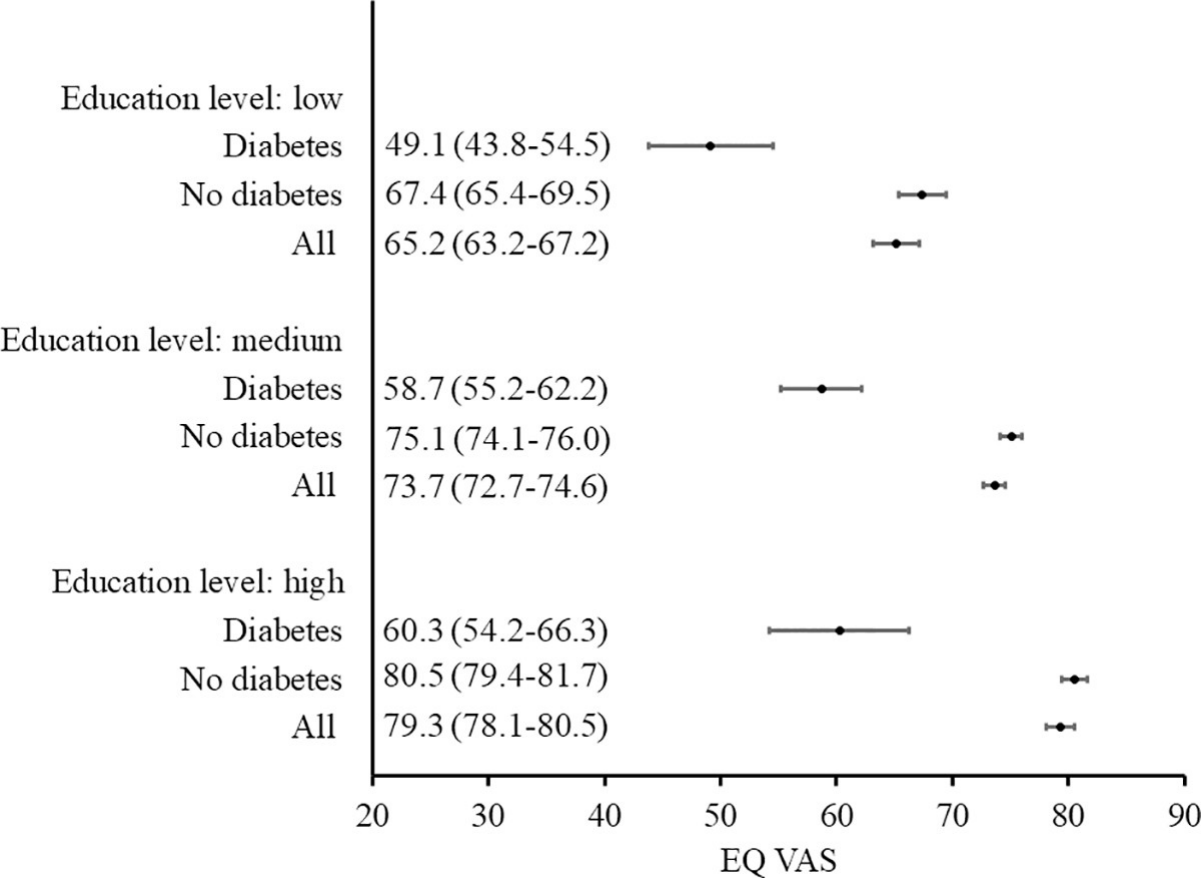
EQ VAS (mean, 95% confidence interval) according to education level: Comparison of respondents with diabetes versus no diabetes and the general population.

The relationship between EQ-5D-5L index or EQ VAS and the sociodemographic characteristics of respondents with diabetes is summarized in Table 5, S1 and S2 Figs. The multivariate analysis showed that the factors independently reducing the quality of life of patients with diabetes (measured with EQ-5D-5L index) were being aged 65 years or above or residing in the provinces of Podlasie or Pomerania, while factor increasing EQ-5D-5L index - secondary or higher education. The subjective HRQoL assessment, measured with EQ VAS, was significantly lower when belonging to older age groups and higher when having greater levels of education. Figs 1 and 2 present 95% confidence intervals for EQ-5D-5L index, according to age group and education level respectively. Figs 3 and 4 present similar analyses for EQ VAS.

### Sociodemographic characteristics and HRQoL in the general society sample

Table 6 presents the relationship between the EQ-5D-5L index or EQ VAS and the sociodemographic characteristics of all respondents in the study. The multivariate analysis indicated that the factors independently improving the quality of life in the general population were secondary or higher education, and factors reducing HRQoL were female sex, belonging to an older age group, being treated because of diabetes with insulin, drugs other than insulin or combination treatment. Respondents diagnosed with diabetes but not treated with drugs showed a decrease in EQ VAS scores, but not in the EQ-5D-5L index. S3 Fig. presents the comparison of limitations within EQ-5D-5L dimensions in respondents with or without diabetes, according to the age group.

**Table 6:** Relation of EQ-5D-5L index and EQ VAS with demographic characteristics of the studied general population sample (n = 2973).

	N (%)	EQ-5D-5L index		EQ VAS	
		Mean (SD)	Multiple linear regression coefficients	Mean (SD)	Multiple linear regression coefficients
**Overall**	2973	0.920 (0.150)		73.5 (20.1)	
**Intercept**			0.957 *		80.20 *
**Gender**					
Male	1390 (46.8)	0.932 (0.131)	-	75.1 (19.6)	-
Female	1390 (46.8)	0.910 (0.163)	-0.0190 *	72.2 (20.5)	-2.33 **
**Age group**					
18–49 years	1503 (50.6)	0.970 (0.074)	-	82.3 (15.9)	-
50–64 years	829 (27.9)	0.906 (0.154)	-0.0485 *	69.0 (19.3)	-11.3 *
65+ years	641 (21.6)	0.822 (0.212)	-0.1178 *	58.7 (19.4)	-19.8 *
**Education level**					
Low	1268 (42.7)	0.888 (0.185)	-	68.5 (21.3)	-
Medium	1089 (36.6)	0.936 (0.121)	0.0313 *	75.6 (19.4)	4.15 *
High	616 (20.7)	0.959 (0.088)	0.0397 *	80.2 (15.6)	6.38 *
**Diabetes and its treatment**					
No diabetes	2718 (91.4)	0.932 (0.130)	-	75.1 (19.2)	-
Diabetes, no drug treatment	62 (2.1)	0.874 (0.208)	-0.0327 (p = 0.0594)	63.0 (20.7)	-7.74 **
Diabetes, drugs other than insulin/combination treatment	137 (4.6)	0.793 (0.252)	-0.0813 *	56.9 (21.2)	-8.30 *
Diabetes, insulin	56 (1.9)	0.719 (0.271)	-0.1485 *	48.5 (21.0)	-15.7 *

## Discussion

We conducted the EQ-5D-5L questionnaire-based survey using a large representative sample of the general population of Poland, and developed quality of life norms for patients with self-reported diabetes. Although diabetes mellitus (DM) leads to a decrease in HRQoL across all age groups, patients with a basic level of education turned out to be a particularly vulnerable subpopulation. The developed normative data can be used in both clinical work and the health technology assessment (HTA) of new anti-diabetic drugs. This is the first study of HRQoL in patients with DM in Poland that is based on the EQ-5D-5L questionnaire.

One of the significant limitations of our study may be the moderate size of the subpopulation of patients who have declared the presence of diabetes. On the other hand, one should bear in mind that in order to identify this group, we approached nearly 3 000 representatives of the general population. The prevalence of self-reported diabetes (8.6%) and self-reported treated diabetes (6.5%) in our study was similar to that observed in the Polish-Norwegian Study (PONS; 8.4%; n = 3 854) [34], NATPOL PLUS in 2002 (6.4%; n = 3 051) [35] and NATPOL 2011 study (6.7%; n = 2 411) [36], which confirms the appropriate selection of the population.

Another limitation of our study results from the method used to conduct it, specifically the limitations associated with a questionnaire survey. Though we ensured the proper recruitment of respondents with the use of stratified sampling, in the study itself the respondents self-declared their diagnosis of diabetes. We did not verify these diagnoses with fasting plasma glucose levels, blood HbA1c levels or by using data from medical records or National Health Fund registers. Nevertheless, this is the approach widely used in epidemiological research, and our results are comparable with numerous studies undertaken on other populations [37–39].

Several issues may be raised in terms of the survey used. In diabetes, diet is often the only therapy in the early stage of the disease. We asked about the diagnosis of diabetes and the usage of medications, but there was no ‘diet’ among treatment options. As we expected respondents’ answers to be less reliable, we did not collect the data on the type of diabetes (type 1, type 2). Still, instead, we obtained the information on the insulin dependence of the condition. Some other data, like self-reported weight and height (allowing calculation of Body Mass Index), disease duration, or the presence of micro and macroangiopathy, could have added valuable information about the health status of the diabetes patients. These data could improve the applicability of the diabetes population norms obtained in this study both as a reference point in clinical assessment and in modelling of the disease in economic evaluations.

The strongest point of our study is clearly the method of sample selection, based on multistage stratified sampling using 65 strata and numbers from the PESEL database. This enabled us to obtain a representative sample of the Polish population in terms of multiple criteria.

A significant number of HRQoL studies among patients with diabetes in Poland have already been published. These have mainly concerned type II diabetes [40–48], with studies on type I diabetes [49] or both types I and II being less common [50–52]. Some of the research focused on precisely defined subpopulations of diabetes patients, such as diabetic foot ulceration [53], neuropathic pain [54, 55], peripheral diabetic neuropathy [56], maturity onset diabetes of the young (MODY) [57], transcatheter aortic valve implantation (TAVI) [58], gestational diabetes [59] or pre-diabetes [60]. The authors willingly use disease-specific questionnaires, including ADDQoL [40, 46, 47, 53], Diabetes Quality of Life—Brief Clinical Inventory (DQL-BCI) [41–43], Diabetes Symptom Checklist-Revised (DSC-R) [41–43], Diabetic Foot Ulcer Scale short form [49] and the PedsQL Diabetes Module 3.0 questionnaire [45]. Concerning generic questionnaires, for Polish patients with diabetes the following were used: SF-36 [36, 39, 48, 49, 52, 61], World Health Organization Quality of Life-Bref (WHOQOL-Bref) [38, 55], and the EQ-5D-3L, which is undoubtedly the most commonly used [36, 41–44, 50, 51, 54].

Polish researchers present a considerable heterogeneity of approaches in seizing the opportunities offered by the EQ-5D framework. Some of them use only one of the available endpoints–EQ VAS [50, 51] or limitations according to dimensions of the questionnaire [36]. Some researchers estimate two outcomes—VAS and HRQoL domains [54] or VAS and EQ-5D index [41–43]. The practice of using the full spectrum of possible results offered by the EQ-5D and calculating all three endpoints is rare [44]. This study is the first Polish survey of HRQoL in diabetes sufferers which employs the new five-level version of the EQ-5D questionnaire.

Both versions of the EQ-5D questionnaire (EQ-5D-3L and EQ-5D-5L) were validated in patients with diabetes [27–29, 62–64]. The EQ-5D-5L seems to be characterized by having a lower ceiling effect, more discriminatory power, and a higher degree of preference among the respondents. Moreover, the conditions for the use of EQ-5D in Poland were developed by the publication of Polish population norms (by age and sex) for both EQ-5D-3L [65] and EQ-5D-5L [66, 67], as well as the release of country-specific value sets reflecting the health preferences of Polish society, for both versions of the questionnaire [29, 68].

In our study, patients with self-reported diabetes, in comparison to the general population, were marked by a higher prevalence of health limitations across all dimensions of the EQ-5D questionnaire. The most significant differences concerned the dimensions of mobility, pain/discomfort and anxiety/depression. A similar hierarchy of affected dimensions was observed when comparing older Chinese patients with type 2 diabetes (T2D) with their age and gender-matched controls [69]. The subjective assessment of the health of Polish respondents with diabetes was significantly lower than in the general population—by 16.9 points on the EQ VAS scale. This difference was smaller than that obtained from data collected in Poland in 2008 (average 18.8 points) [44], but higher than that observed in the German population (12.5 points) [70]. In Poland, respondents with diabetes, compared to respondents from the general population, had an EQ-5D-5L index value that was 0.123 lower. This difference was higher than that observed in Japan (0.090) [71], China (0.072) [64] or Canada—in the provinces of Quebec and Alberta (0.084 and 0.040) [72, 73]. The use of EQ-5D allows international comparisons to be readily performed.

## Conclusions

The paper reports EQ-5D-5L normative data for Polish patients with self-reported diabetes, based on a national representative sample. These results may be used in outcome measurement in clinical care, as well as in economic analyses and health technology assessment reports for new anti-diabetic drugs.

## Supporting information

S1 Fig Mean EQ VAS in respondents with self-reported diabetes according to voivodeship. (TIF)

S2 Fig Mean EQ-5D-5L index in respondents with self-reported diabetes according to voivodeship. (TIF)

S3 Fig Cumulative percentage of limitations within EQ-5D-5L dimensions in patients with diabetes compared to respondents without diabetes, according to age group. (DOCX)

S1 File Study anonymized dataset. (XLSX)
