# Supplementary figures and images for: EQ-5D-5L-based quality of life normative data for patients with self-reported diabetes in Poland
Source: PLoS One. 2021 Sep 29;16(9):e0257998. doi: 10.1371/journal.pone.0257998 (PMC8480847; doi:10.1371/journal.pone.0257998)

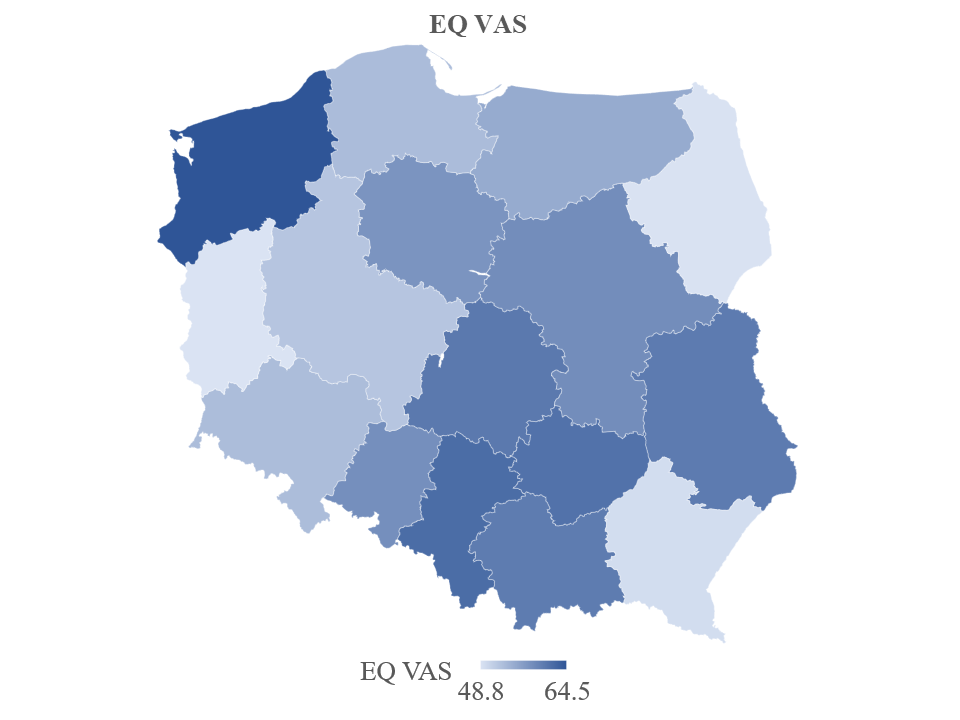

Supplement: S1 Fig — (TIF) [file pone.0257998.s001.tif]

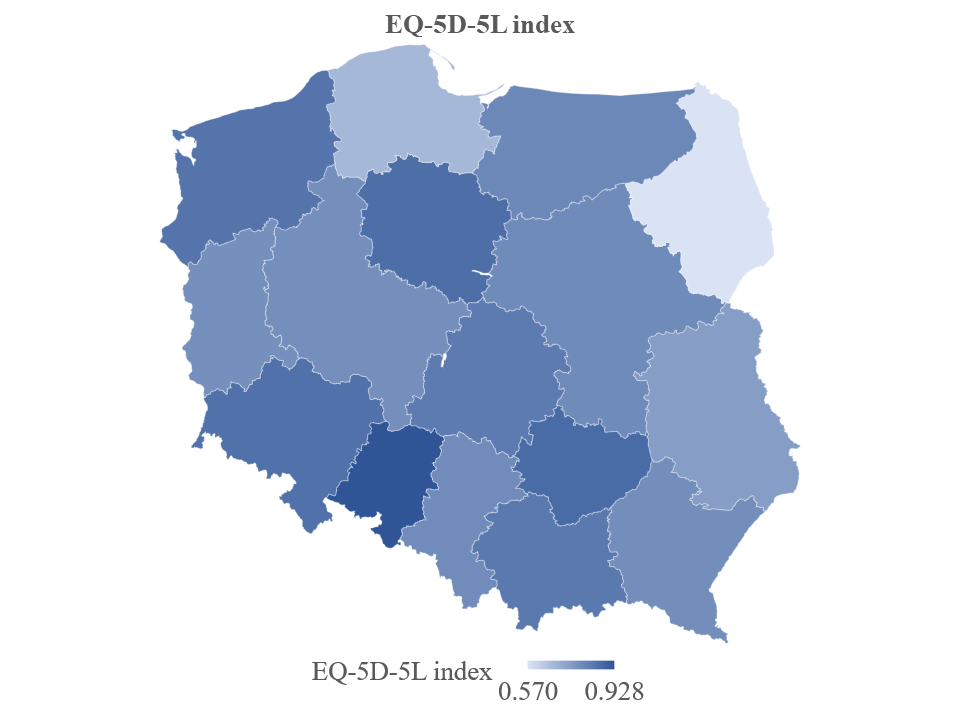

Supplement: S2 Fig — (TIF) [file pone.0257998.s002.tif]

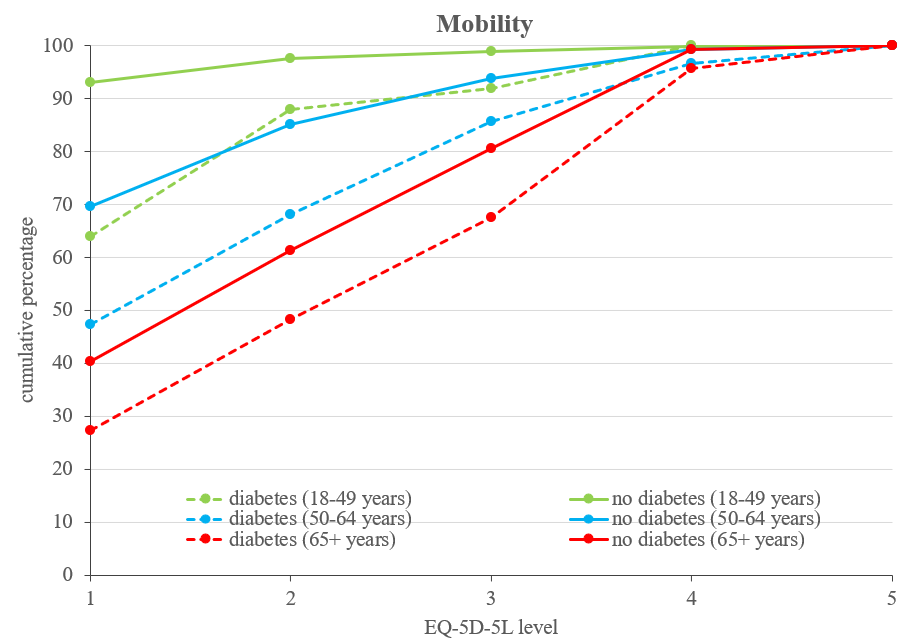


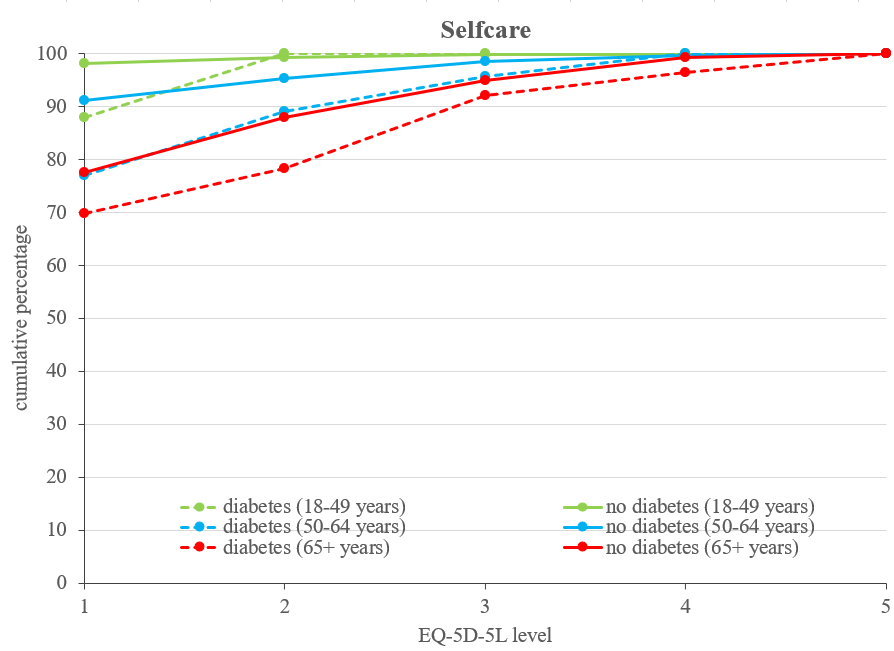


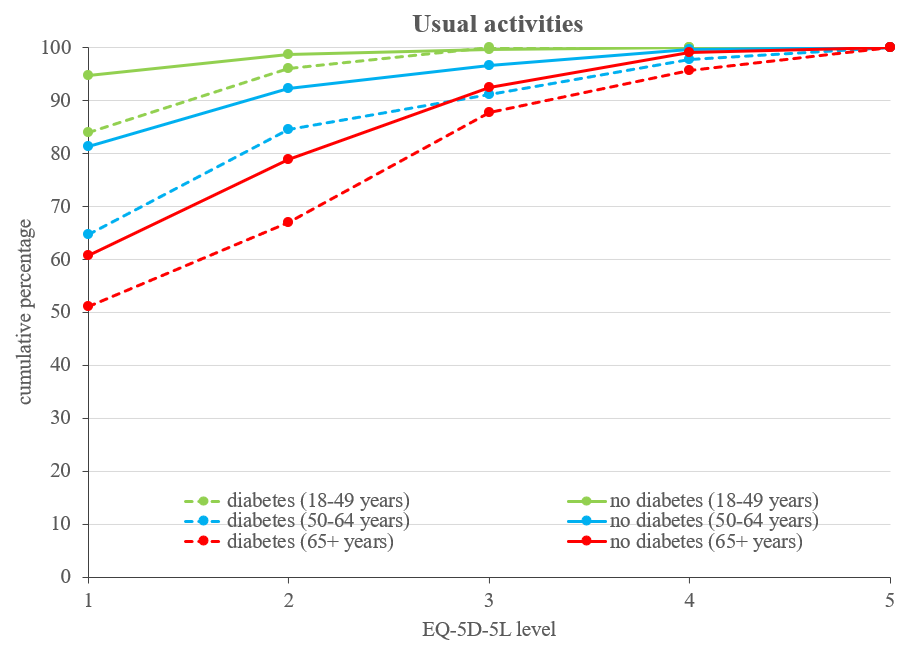


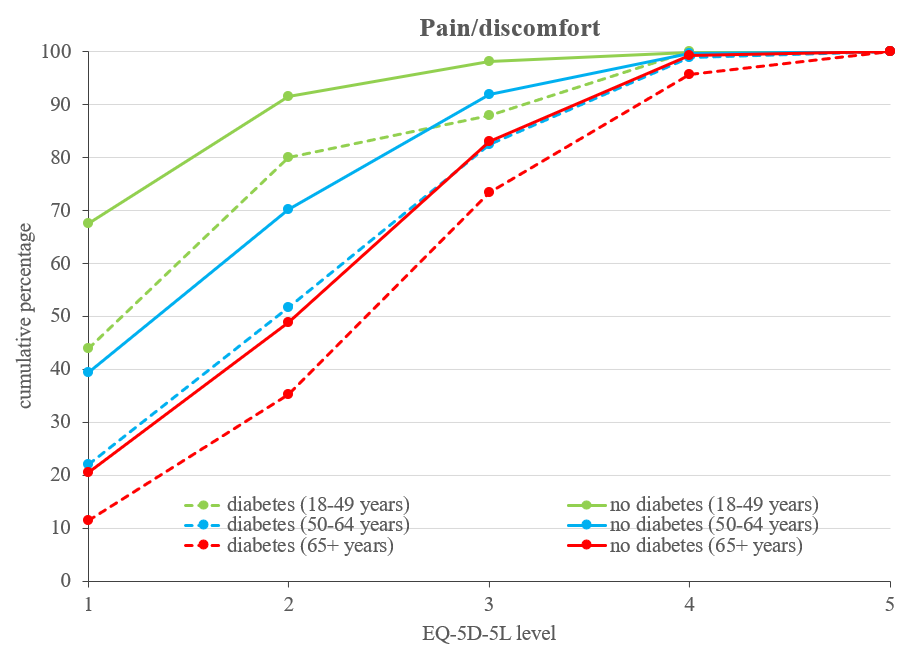


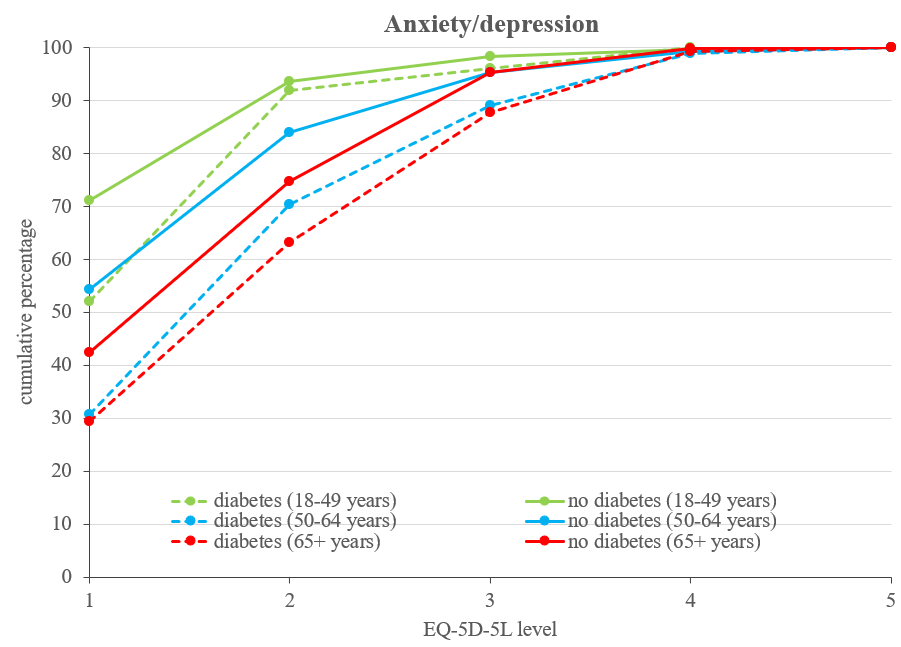

Supplement: S3 Fig — (DOCX) [file pone.0257998.s003.docx]
